# Prevalence of the *GJB2 *IVS1+1G >A mutation in Chinese hearing loss patients with monoallelic pathogenic mutation in the coding region of *GJB2*

**DOI:** 10.1186/1479-5876-8-127

**Published:** 2010-12-02

**Authors:** Yongyi Yuan, Fei Yu, Guojian Wang, Shasha Huang, Ruili Yu, Xin Zhang, Deliang Huang, Dongyi Han, Pu Dai

**Affiliations:** 1 Department of Otolaryngology, PLA General Hospital, Beijing, People's Republic of China

## Abstract

**Background:**

Mutations in the GJB2 gene are the most common cause of nonsyndromic recessive hearing loss in China. In about 6% of Chinese patients with severe to profound sensorineural hearing impairment, only monoallelic *GJB2 *mutations known to be either recessive or of unclear pathogenicity have been identified. This paper reports the prevalence of the *GJB2 *IVS1+1G>A mutation in a population of Chinese hearing loss patients with monoallelic pathogenic mutation in the coding region of *GJB2*.

**Methods:**

Two hundred and twelve patients, screened from 7133 cases of nonsyndromic hearing loss in China, with monoallelic mutation (mainly frameshift and nonsense mutation) in the coding region of *GJB2 *were examined for the *GJB2 *IVS1+1G>A mutation and mutations in the promoter region of this gene. Two hundred and sixty-two nonsyndromic hearing loss patients without *GJB2 *mutation and 105 controls with normal hearing were also tested for the *GJB2 *IVS1+1G>A mutation by sequencing.

**Results:**

Four patients with monoallelic mutation in the coding region of *GJB2 *were found carrying the *GJB2 *IVS1+1G>A mutation on the opposite allele. One patient with the *GJB2 *c.235delC mutation carried one variant, -3175 C>T, in exon 1 of *GJB2*. Neither *GJB2 *IVS1+1G>A mutation nor any variant in exon 1 of *GJB2 *was found in the 262 nonsyndromic hearing loss patients without *GJB2 *mutation or in the 105 normal hearing controls.

**Conclusion:**

Testing for the *GJB2 *IVS 1+1 G to A mutation explained deafness in 1.89% of Chinese *GJB2 *monoallelic patients, and it should be included in routine testing of patients with *GJB2 *monoallelic pathogenic mutation.

## Introduction

Hereditary hearing loss is a genetically heterogeneous disorder in humans, with an incidence rate of approximately 1 in 1000 children [1]. Nonsyndromic deafness accounts for 60-70% of cases of inherited hearing impairment and involves 114 loci and 55 different genes with autosomal dominant (DFNA), autosomal recessive (DNFB), X-linked (DFN), and maternal inheritance patterns [2]. The most common causes of nonsyndromic autosomal recessive hearing loss are mutations in connexin 26, a gap-junction protein encoded by the GJB2 gene [3-10].

To date, more than 150 mutations, polymorphisms, and unclassified variants have been described in the GJB2 gene, which account for the molecular etiology of 10-50% of patients with nonsyndromic hearing impairment http://davinci.crg.es/deafness. Therefore, *GJB2 *is normally the first gene to be tested in patients with hearing loss. In China, the ratio of patients carrying mutations in the coding exons of *GJB2 *is 21% (biallelic, 14.9%; monoallelic, 6.1%) [11]. However, few studies have examined the noncoding exon 1 of *GJB2 *in Chinese hearing-impaired patients, and even fewer studies have investigated the promoter region of this gene. The results of *GJB2 *screening performed to date have indicated that a substantial fraction of patients (6-15%) carry only one pathogenic mutation in the GJB2 gene with either recessive or unclear pathogenicity, despite direct sequencing of the entire coding region of the gene [12-14]. The ratio of a 309-kb deletion involving the GJB6 gene, now called del(GJB6-D13S1830), was shown to be the second causal mutation in these monoallelic heterozygous patients in Spain and France [15,16]. Previously, we tested Chinese patients with only one monoallelic mutation in the coding region of *GJB2 *for the presence of this mutation, but the results indicated this to be a very rare cause of hearing loss in the Chinese population, and this is not a major additional factor in our monoallelic patients (unpublished). Similar results have also been reported in Austria and the Czech Republic [17,18]. The splice site mutation IVS1+1G>A, also called the -3170 G>A mutation, in the GJB2 gene was originally reported by Denoyelle *et al*. [19]. This splice site mutation has been found in several populations [20-26] and is predicted to disrupt splicing, yielding no detectable mRNA [20]. Not all genetic laboratories routinely test for this mutation, which lies outside the coding region of the GJB2 gene. This study focused on clarifying the impact of *GJB2 *IVS1+1G>A mutation and the promoter region of this gene among Chinese patients with hearing loss, especially those with pathogenic mutation in only one allele of the GJB2 gene coding region.

## Materials and methods

### Patients and DNA samples

A total of 212 deaf subjects with monoallelic mutation in the coding region of *GJB2 *and 262 unrelated nonsyndromic hearing loss patients without *GJB2 *mutation from unrelated families were included in this study. The 212 deaf subjects with monoallelic mutation, mainly frameshift and nonsense mutations, in the coding region of *GJB2 *were screened from a total of 7133 nonsyndromic hearing loss cases in China (Table 1). Of the 7133 cases, 3433 were collected from 28 different regions, covering 90% of the provinces in China; 3700 were patients of the Genetic Testing Center for Deafness, PLA General Hospital, during the period from March 2002 to December 2010. The majority of the 7133 patients were Han Chinese (6540), followed by Southwest Chinese minorities (134, including Buyi, Hani, Yao, Yi, Bai, Wa, Miao, Dong, Tujia, Lahu, Dai, Bulang, Sala, etc.), Tibetan (123), Hui (113), minorities from the Xinjiang Uyghur Autonomous Region (77), Mongolian (63), Maan (51), Chuang (27), and Korean (5). Ethnic subgroup designations were based on permanent residency documentation.

**Table 1:** GJB2 IVS1+1G>A mutation in Chinese hearing loss patients with monoallelic pathogenic mutation in GJB2

Allele 1			Allele 2			
Exon 2			Exon 1 or splice site			

Nucleotide change	Consequence or amino acid change	Category	Nucleotide change	Consequence or amino acid change	Category	Number of patients

c.235delC	Frameshift mutation	pathogenic	IVS1+1G>A	Splicing site mutation	pathogenic	2
c.35delG	Frameshift mutation	pathogenic	IVS1+1G>A	Splicing site mutation	pathogenic	1
c.9G>A/c.11G>A	W3X/G4D	pathogenic/pathogenic	IVS1+1G>A	Splicing site mutation	pathogenic	1
c.235delC	Frameshift mutation	pathogenic	c.-3175C>T	Non-coding	Not determined	1
c.235delC	Frameshift mutation	pathogenic				161
c.299delAT	Frameshift mutation	pathogenic				24
c.176del16bp	Frameshift mutation	pathogenic				6
c.35delG	Frameshift mutation	pathogenic				4
c.424_426 delTTC	Frameshift mutation	pathogenic				4
c.9G>A	W3X	pathogenic				1
c.512insAACG	Frameshift mutation	pathogenic				2
c.605ins46	Frameshift mutation	pathogenic				2
c.155_158delTCTG	Frameshift mutation	pathogenic				1
c.35insG	Frameshift mutation	pathogenic				2
**Total**						**212**

The 212 deaf patients consisted of 123 males and 90 females from 0.2 to 67 years old, with an average age of 5.41 ± 1.78 years. Ethnically, the patients consisted of 196 Han, 4 Hui, 3 Uygur, 3 Mongolian, 2 Tibetan, 2 Maan, 1 Miao, 1 Chuang, and 1 Buyi Chinese.

The 262 unrelated nonsyndromic hearing loss patients without *GJB2 *coding region mutation were selected randomly from patients of the Genetic Testing Center for Deafness, PLA General Hospital, during the year 2007. This cohort consisted of 147 males and 115 females from 2 to 46 years old with an average age of 4.52 ± 1.16 years, and ethnically, they were all Han Chinese.

The study protocol was performed with the approval of the Ethics Committee of the Chinese PLA General Hospital. Informed consent was obtained from all subjects prior to blood sampling. The parents of pediatric patients were interviewed with regard to age of onset, family history, mother's health during pregnancy, and patient's clinical history, including infection, possible head or brain injury, and the use of aminoglycoside antibiotics. All subjects showed moderate to profound bilateral sensorineural hearing impairment on audiograms. Careful medical examinations revealed no clinical features other than hearing impairment. DNA was extracted from the peripheral blood leukocytes of the 474 (212 + 262) patients with nonsyndromic hearing loss and 105 controls with normal hearing using a commercially available DNA extraction kit (Watson Biotechnologies Inc., Shanghai, China).

### Mutational analysis

The coding exon (exon 2) and flanking intronic regions of GJB2 gene were amplified by PCR with the primers F (5'TTG-GTG-TTT-GCT-CAG-GAA-GA-3') and R (5'GGC-CTA-CAG-GGG-TTT-CAA-AT-3') in all 7133 nonsyndromic hearing loss cases. The *GJB2 *exon 1, its flanking donor splice site and the *GJB2 *basal promoter were amplified with the primers F (5'CTC-ATG-GGG-GCT-CAA-AGG-AAC-TAG-GAG-ATC-GG-3') and R (5'GGG-GCT-GGA-CCA-ACA-CAC-GTC-CTT-GGG-3') in all subjects with monoallelic mutation in the coding region of *GJB2*, 262 unrelated nonsyndromic hearing loss patients without *GJB2 *mutation, and 105 normal controls.

All the patients and controls were also tested for *GJB6 *309-kb deletion and the coding exon of *GJB6*. The presence of the 309-kb deletion of *GJB6 *was analyzed by PCR [15,27]. A positive control (provided by Balin Wu, Department of Laboratory Medicine, Children's Hospital Boston and Harvard Medical School, Boston, MA) was used for detection of GJB6 gene deletions. The coding exon of GJB6 was amplified with the primers F (5' TTG-GCT-TCA-GTC-TGT-AAT-ATC-ACC-3') and R (5' TCA-TTT-ACA-AAC-TCT-TCA-GGC-TAC-AG-3'). All the PCR products were purified on Qia-quick spin columns (Qiagen, Valencia, CA) and sequenced using a BigDye Terminator Cycle Sequencing kit (version v.3.1) and ABI 3130 automated DNA sequencer (Applied Biosystems, Foster City, CA) with sequence-analysis software (Sequencing Analysis version v.3.7) according to the manufacturer's protocol.

Mitochondrial *12S rRNA *and *SLC26A4 *were also sequenced in the 262 unrelated nonsyndromic hearing loss patients without *GJB2 *coding region mutation. DNA sequence analysis of mitochondrial *12S rRNA *and *SLC26A4 *were performed by PCR amplification of the coding exons plus approximated 50-100 bp of the flanking intron regions followed by Big Dye sequencing and analysis using ABI 3100 DNA sequencing machine (ABI, Foster City, USA.) and ABI 3100 Analysis Software v.3.7 NT according to manufacturer's procedures.

## Results

### Hearing phenotype

Deafness in 10.8%(767/7133) of the 7133 nonsyndromic hearing loss patients is postlingual and in 89.2% (6366/7133) is preligual. The percent of postlingual hearing loss in the 212 nonsyndromic hearing loss patients group with monoallelic mutation in the coding region of *GJB2 *is 6.6%(14/212) and that of preligual is 93.4% (198/212). The percent of postlingual hearing loss in the 262 nonsyndromic hearing loss patients group without *GJB2 *coding region mutation is 8%(21/262) and that of preligual is 92% (241/262). The average onset age of postlingual hearing loss in the 7133 patient cohort is 3.19 ± 1.56 years, and that age in the 212 patient group with monoallelic mutation in the coding region of *GJB2 *and the 262 patient group without *GJB2 *coding region mutation is 2.78 ± 1.06 years and 3.04 ± 2.39 years, respectively.

All of the 212 unrelated patients with monoallelic *GJB2 *coding region mutation as well as the 262 unrelated nonsyndromic hearing loss patients without *GJB2 *coding region mutation showed bilateral moderate to profound sensorineural hearing loss. None of the patients in this study showed clinical signs in any other organs except hearing impairment.

### Genetic results

By direct sequencing analysis of 7133 Chinese patients with hearing impairment, we found 212 unrelated patients with monoallelic *GJB2 *coding region mutation. All of the 212 patients carried frameshift or nonsense pathogenic mutations leading to insertion of a premature stop codon. The detailed genotypes of the 212 patients are shown in Table 1. We detected four patients carrying the IVS1+1G>A mutation in the heterozygous state in addition to their already known c.235delC, c.35delG, and W3X mutations, respectively [two of the patients both carry the c.235delC mutation]. One novel variant in the *GJB2 *exon 1, -3175 C>T, was detected in a patient with 235delC mutation. No mutations or variants in the *GJB2 *basal promoter region were found in this study. In three of the compound heterozygotes carrying IVS1+1G>A and pathogenic mutation in the exon 2 of *GJB2*, the separate segregation of each allele was confirmed in either the parents or patients' siblings (Table 2). We could not obtain pedigree blood samples in only one patient with *GJB2 *IVS1+1G>A/35delG mutation. This patient was of the Uygur ethnic minority from Xinjiang Uyghur Autonomous Region. In the patient whose genotype is IVS1+1G>A,c.11G>A(G4D)/c.9G>A(W3X), we confirmed the result by the analysis of the proband's parents' two alleles. We found that the father carried both IVS1+1G>A and c.11G>A(G4D) in one allele and the mother carried c.9G>A(W3X) in one allele, while the opposite alleles of the parents were both wild-type. After inclusion of the IVS1+1G>A mutation in our detection procedure, the percentage of individuals with bilateral sensorineural hearing loss with only one monoallelic frameshift or nonsense mutation in *GJB2 *decreased from 2.97% (212/7133) to 2.92% (208/7133).

**Table 2:** Mutations of GJB2 Exon 1 in Chinese hearing loss patients with monoallelic pathogenic mutation in GJB2

No.	Age	Family history	Ethnicity	Genotype of the proband (EXON 1/EXON 2)	Genotype of the proband's father	Genotype of the proband's mother	Genotype of the proband's siblings
							
1	21	No	Han	IVS1+1G>A/c.235delC	wt/c.235delC	IVS1+1G>A/wt	wt/wt
2	2	No	Han	IVS1+1G>A/c.235delC	wt/c.235delC	IVS1+1G>A/wt	
3	1	No	Han	IVS1+1G>A,c.11G>A(G4D)/c.9G>A(W3X)	IVS1+1G>A, c.11G>A(G4D)/wt	wt/c.9G>A(W3X)	
4	23	No	Uyghur	IVS1+1G>A/c.35delG	No blood sample	No blood sample	No blood sample
5	8	No	Han	c.-3175C>T/c.235delC	c.-3175C>T/wt	No blood sample	

Among the 262 patients without *GJB2 *mutation, four carried the mitochondrial *12S rRNA *A1555G mutation, and 19 carried *SLC26A4 *mutations and were diagnosed as having enlarged vestibular aqueduct by temporal CT scan. None of these patients was found to carry the *GJB2 *IVS1+1G>A mutation. One patient was shown to carry the *GJB6 *c.404C>A mutation (T135K), and this patient had no mutation in mitochondrial *12S rRNA *or *SLC26A4*. This patient was of the Uygur ethnic minority from Xinjiang Uyghur Autonomous Region.

In the control group, we detected two c.235delC and one c.299delAT heterozygotes, representing 3%, which coincided with our previous results in a different control cohort [11]. No *GJB2 *IVS1+1G>A mutation was detected in the control group. A *GJB6 *variant, c.446 C>T mutation (A149V), was detected in an individual of the Uygur ethnic minority.

We did not find the 309-kb deletion of *GJB6 *in any of the 212 patients with monoallelic *GJB2 *coding region mutation or in any of the 105 samples from normal hearing controls with no history of hearing loss.

## Discussion

The GJB2 gene is composed of two exons separated by an intron, and the coding region is entirely contained in exon 2. The basal promoter activity resides in the first 128 nucleotides upstream of the transcription start point (TSP) and has two GC boxes, at positions 281 and 293 from the TSP, which are important for transcription [28]. Most of the *GJB2 *sequence variations described to date are localized in the coding region, and only a few have been reported in noncoding regions of the gene [19,23,29-31]. Mutational screening performed to date has usually focused on the coding region. *GJB2 *is responsible for up to 21% of cases of deafness in the Chinese population [12]. The most common mutation is a frameshift mutation due to deletion of a single cytosine at position 235 (235delC). The four most prevalent mutations: c.235delC, c.299_c.300delAT, c.176_c.191del16, and c.35delG, account for 88.0% of all mutant *GJB2 *alleles identified in China [11].

Sequence analysis of the GJB2 gene in subjects with autosomal recessive hearing impairment has revealed a puzzling problem in that a large proportion of patients (6-15%) carry only one mutant allele [14-17]. Some of these families showed clear evidence of linkage to the DFNB1 locus, which contains two genes, *GJB2 *and *GJB6 *[3]. Further analysis demonstrated a 309-kb deletion, truncating the GJB6 gene, encoding connexin 30, near *GJB2 *in heterozygous affected subjects [18,19]. We had tested Chinese patients with only one monoallelic mutation in the coding region of *GJB2 *for the presence of this deletion, but it was shown to be a very rare cause of deafness in the Chinese population. Similar results in populations in Turkey, Iran, Austria, Taiwan, China, Poland, and the Altai Republic have also been reported [25,32-39]. Cases with one pathogenic mutation in the GJB2 gene may have another as yet unidentified pathogenic mutation in the promoter region or other noncoding regions of *GJB2*.

To evaluate the impact of the IVS1+1G>A splice-site mutation and the basal promoter region in the noncoding part of the GJB2 gene among Chinese patients, we initially carried the sequencing of *GJB2 *exon1 among 851 deaf individuals from Central China and no mutation was found[11], which suggested very low detection rate of *GJB2 *exon1 mutation among Chinese deaf population. Thus we began to collect and test all available nonsyndromic hearing loss patients with only one monoallelic pathogenic mutation in the coding part of *GJB2*. By sequencing exon 1 and the basal promoter region of the GJB2 gene in 212 Chinese patients with *GJB2 *monoallelic mutation, we identified four patients carrying the IVS1+1G>A mutation. Testing for this mutation explained deafness in 1.89% of Chinese *GJB2 *monoallelic patients. This ratio is significantly lower than the value of 45% in Czech patients with one pathogenic mutation in *GJB2 *[40] and 23.40% of Hungarian patients carrying a mutation in only one allele of the coding region of the GJB2 gene [41]. It is also lower than the value of 4.6% among Brazilian patients with one pathogenic *GJB2 *mutation [42]. The percentage of the IVS1+1G>A mutation was 1.85% (4/216) of mutant alleles in our patient cohort, while in the Kurdish deaf population this percentage is 9.4%(3/32)[26], significantly higher than the Chinese population. As for the Mongolian population, the frequency of deaf probands carrying two *GJB2 *pathogenic mutations was 4.5%[43], significantly lower than that (14.9%) in the Chinese deaf population and the mutation spectrums of *GJB2 *is also different from that in China. The most common mutation in *GJB2 *was IVS1+1G to A with an allele frequency of 3.5%[43] in the Mongolian deaf population. While c.235delC was the most common mutation in the Chinese deaf population with an allele frequency of 12.34%[11], significantly higher than that in the Mongolian deaf population which was 1.5%[43]. The differences between the two Asian neighboring countries may lie in two aspects: a) the genetic background of the two races varies. b) in our study IVS1 +1G to A mutation was only screened in hearing loss patients with monoallelic mutation (mainly frameshift and nonsense mutation) in the coding region of *GJB2*. These observations indicate that the carrying rate of *GJB2 *IVS1+1G>A mutation varies among different races. We also tested the IVS1+1G>A mutation in 262 unrelated nonsyndromic hearing loss patients without *GJB2 *ORF mutation and 105 normal controls, but neither homozygous IVS1+1G>A mutation nor heterozygous IVS1+1G>A mutation was found. The IVS1+1G>A mutation may account for the genetic etiology only in patients with *GJB2 *monoallelic pathogenic mutation in the Chinese deaf population, which suggests that the frequency of IVS1+1G>A mutation is very low in Chinese population.

Matos *et al*. [44] reported a *GJB2 *mutation, -3438C>T, located in the basal promoter of the gene, in *trans *with V84M, in a patient with profound hearing impairment. They verified that the -3438C>T mutation can abolish the basal promoter activity of *GJB2*. Although we extended mutational screening to regions of *GJB2 *exon 1, its flanking donor splice site, and the *GJB2 *basal promoter, we found no other mutation except one c.-3175C>T variant in exon 1 and four heterozygous IVS1+1G>A mutations. As the variant, c.-3175C>T, is in the noncoding region, it was taken to be nonpathogenic.

There are two reasons that the percentage of monoallelic mutation in the GJB2 gene in our cohort was lower than our previously reported data (6%) [11], as follows.

a) In this study, we only counted pathogenic mutations, frameshift mutations, and nonsense pathogenic mutations; if all the missense mutations which was not found or the carrier rate was significantly low in the normal hearing controls, were calculated, the rate was increased to 5.5%.

b) Additionally, about 13% of patients had moderate hearing loss, whereas all the patients in our previous study [11] showed severe to profound hearing impairment.

Through genotype and phenotype analysis in 1093 cases of unrelated, nonsyndromic Chinese individuals with hearing loss, *GJB2 *mutations were detected in 24.67% (130/527) of patients with bilateral profound hearing loss, 22.33% (44/197) with bilateral severe hearing loss, 14.33% (42/293) with bilateral moderate hearing loss, and 6.58% (5/76) with bilateral mild hearing loss (unpublished data). The differences between the severe to profound hearing loss group and the mild to moderate hearing loss group were statistically significant. In this patient group, the total percentage of *GJB2 *mutations in all the 1093 cases is 20.22%(221/1093), similar to that in our previous study[11]. Additionally, patients in the above two cohorts didn't overlap.

There are three possible explanations for the failure to detect a second mutant allele in the 208 cases in the present study.

a) The second mutant allele has not yet been identified due to the location of mutations deep in introns that were not sequenced.

b) It is possible that a digenic pattern of inheritance is responsible for these cases. Therefore, the second mutation may be a connexin gene other than *GJB6 *or may involve another gene, the product of which interacts with connexin 26. Clearly, this hypothesis can not be verified until the other mutant alleles have been found.

c) Part of these heterozygous probands are simply carriers, and their hearing impairment may have other causes.

## Conclusion

Testing for the *GJB2 *IVS 1+1 G to A mutation explained deafness in 1.89% of Chinese *GJB2 *monoallelic patients. Although the percentage is not as high as those in Western and Mongolian populations, it can still serve as a routine testing point in patients with *GJB2 *monoallelic pathogenic mutation in China.

## Conflict of interest statement

The authors declare that they have no competing interests.

## Authors' contributions

YY, FY, GW, SH, RY and XZ carried out the molecular genetic studies and participated in sequence alignment. YY drafted the manuscript. DeHu and DoHa participated in the design of the study. PD conceived the study, participated in its design and coordination, and helped draft the manuscript. All authors have read and approved the final manuscript.
